# The polymorphisms of *FGFR2* and *MGAT5* affect the susceptibility to COPD in the Chinese people

**DOI:** 10.1186/s12890-021-01498-3

**Published:** 2021-04-20

**Authors:** Xiaobo Li, Guangyu Zhou, Xiaobo Tian, Fei Chen, Guoyao Li, Yipeng Ding

**Affiliations:** 1 Department of General Practice, People’s Hospital of Wanning, Wanning, 571500 Hainan China; 2 Department of Nursing, People’s Hospital of Wanning, Wanning, 571500 Hainan China; 3 Department of Medical, People’s Hospital of Wanning, Wanning, 571500 Hainan China; 4 Nanyang Branch of Wencheng Health Center of Wenchang City, Wenchang, 571399 Hainan China; 5 Department of Science and Education Department, Hainan General Hospital, Hainan Affiliated Hospital of Hainan Medical University, Haikou, 570311 Hainan China; 6 Department of General Practice, Hainan General Hospital, Hainan Affiliated Hospital of Hainan Medical University, No. 19, Xinhua Road, Xiuying District, Haikou, 570311 Hainan China

**Keywords:** Chronic obstructive pulmonary disease, *FGFR2*, *MGAT5*, Genetic polymorphism

## Abstract

**Background:**

Chronic obstructive pulmonary disease (COPD) is characterized by incomplete reversible airflow limitation and chronic inflammatory response lesions. This study mainly explored whether *FGFR2* and *MGAT5* polymorphisms affected the risk of COPD in the Chinese people.

**Methods:**

Five variants in *FGFR2* and *MGAT5* were chosen and genotyped using Agena MassARRAY platform from 315 COPD patients and 314 healthy controls. The correlation of *FGFR2* and *MGAT5* with COPD susceptibility was evaluated with odds ratio (OR) and 95% confidence interval (CI) via logistic regression.

**Results:**

We found rs2420915 enhanced the risk of COPD, while rs6430491, rs2593704 reduced the susceptibility of COPD (*p* < 0.05). Rs2420915 could promote the incidence of COPD in the elderly and nonsmokers. Rs1907240 and rs2257129 also increased the susceptibility to COPD in nonsmokers (*p* < 0.05). *MGAT5-*rs2593704 played a protective role in COPD development in different subgroups (age ≤ 70, male, smokers, and individuals with BMI ≤ 24 kg/m^2^). Meanwhile, rs6430491 was linked with a lower risk of COPD in nonsmoking and BMI ≤ 24 kg/m^2^ subgroups.

**Conclusions:**

We concluded that *FGFR2* and *MGAT5* genetic polymorphisms are correlated with the risk of COPD in the Chinese people. These data underscored the important role of *FGFR2* and *MGAT5* gene in the occurrence of COPD and provided new biomarkers for COPD treatment.

*Trial registration*: NA.

**Supplementary Information:**

The online version contains supplementary material available at 10.1186/s12890-021-01498-3.

## Background

Chronic obstructive pulmonary disease (COPD) is a common chronic disease of the respiratory system, which is mainly characterized by incomplete reversible airflow limitation and chronic inflammatory response lesions. The physiopathology of COPD were airflow limitation, gas exchange abnormalities, degeneration, necrosis and ulceration of bronchial epithelial cell, excessive expansion of lung, pale appearance and bullae of different sizes on the surface. The main clinical manifestations are cough, sputum, dyspnea, and decreased exercise endurance, which can eventually lead to pulmonary heart disease and respiratory failure. COPD has the characteristics of high morbidity, mortality and disability among the elderly. Epidemiological investigation has shown that the incidence of COPD in the Chinese population over 40 years old is approximately 13.7%, which is the third most fatal disease in the world [1]. In addition, it is reported that COPD can seriously affect the quality of life of patients [2]. According to the World Bank report, COPD is expected to account for the fifth-largest economic burden of disease worldwide by 2020. Therefore, it is important to explore the pathogenesis and etiology of COPD.

Many investigations have demonstrated that tobacco smoking is an important risk factor for COPD development [3]. Nevertheless, only 10%-20% of smokers develop COPD, and 30% of nonsmokers suffer from COPD, suggesting genetic background plays a crucial role in COPD development [4, 5]. A genome-wide study found that *MAN2B, DHX15* gene were associated with COPD susceptibility in multi-ethnic populations [6]. Du et al. also showed the genetic variants of *GSTP1*, *HO-1*, and *SOD-3* were correlated with COPD susceptibility [7]. Besides, other studies have been reported that *PDE4D*, *FAM13A*, *CYP2B6* gene polymorphisms may exert effects on COPD susceptibility [8–10]. These findings highlighted the important role of genetic polymorphisms in the occurrence of COPD.

Fibroblast growth factor receptor 2 (*FGFR2*) is one of the members of the fibroblast growth factor receptor (*FGFR*) family, and four types, namely *FGFR1*, *FGFR2*, *FGFR3*, and *FGFR4*, have been identified*.* The *FGFR* family members are involved in a variety of physiological processes, including cell growth and migration [11]. It is reported that *FGFR2* participated in lung development and it is considered as a therapeutic target for lung cancer [12, 13]. Dorry et al. [14] have shown that alveolar epithelial cell-specific *FGFR2* was critical for survival in response to bleomucin-induced lung injury. Jieming et al. [15] found that *FGFR2* mutants could alleviate pulmonary fibrosis of alveolar epithelial type II cells through FGF-2. These lines of evidence have led us to believe that *FGFR2* may be involved in the development of lung-related disease. Nevertheless, the role of FGFR2 gene in COPD has been poorly studied.

N-acetylglucosaminyltransferaseV (*MGAT5*), also known as Gnt-V, catalyzes the formation of β-1,6-branched N-glycans that promote surface retention of glycoproteins [16]. *MGAT5* has been reported to be involved in the proliferation, adhesion, invasion and metastasis of tumor cells [17]. Studies found that *MGAT5* was highly expressed in pulmonary adenocarcinoma cells and its silence suppressed cell growth [18, 19]. In addition, Elek and colleagues illustrated that *MGAT5-*rs34944508 was significantly correlated with lung cancer risk [20]. These findings suggested that *MGAT5* could play a key role in lung disease development. However, little is known about the role of *MGAT5* in COPD development.

In this case–control study, we explored whether *FGFR2* and *MGAT5* genetic mutants influence the occurrence of COPD. We identified and genotyped five single nucleotide polymorphisms (SNPs) from *FGFR2* and *MGAT5* to evaluate the association of SNPs with COPD susceptibility. This will provide new ideas for understanding the pathological mechanism of COPD.

## Methods

### Study population

Our research recruited 629 subjects (315 COPD patients and 314 healthy controls) from Hainan General Hospital. Based on the Global Initiative for Chronic Obstructive Lung Disease criteria, individuals were diagnosed with COPD with the ratio of forced expiratory volume in 1 s (FEV_1_) /forced vital capacity (FVC) < 70% and FEV1 < 80% predicted. COPD patients with a history of bronchial asthma, tuberculosis, lung cancer, and other serious diseases were not included in this study. Controls with healthy subjects without lung dysfunction, no lung-related diseases, other chronic diseases and disorders, and severe endocrine, metabolic, and nutritional disorders from the health checkup in the same hospital during the same period. Clinical characteristics of the study subjects were collected by medical records and questionnaires, including smoking and body mass index (BMI), complications, wheeze, gasp, chest distress and respiratory infection. This study protocol received approval by the Ethics Committee of Hainan General Hospital and conformed to the declarations of Helsinki. And we also got informed consent signed by all participants.

### Genotyping

Five SNPs (rs2420915, rs1907240, rs2257129, rs6430491, rs2593704) were identified and genotyped. All SNPs had a minor allele frequency in the Chinese Han Beijing population. Genomic DNA was extracted from whole blood using a DNA extraction kit (GoldMag Co. Ltd, Xi’an, China) and its concentration was detected by NanoDrop 2000 (Thermo Scientific, Waltham, USA). We applied the Agena MassARRAY platform to genotype. Data analysis and management using Agena Typer 4.0 software.

### Statistical analysis

We applied for student *t*-test and χ^2^ test to assess the difference in age and gender between the cases and the control group. The Hardy–Weinberg equilibrium (HWE) of the control group was calculated by *χ*^2^ test. The relationship between genetic variants with COPD risk was evaluated with odds ratio (OR) and 95% confidence interval (CI) by logistic regression analysis. Haploview software and PLINK software were used for Haploview analysis and linkage disequilibrium [21, 22]. *P* value < 0.05 was considered statistically significant.

## Results

### Characteristics of participants

The demographic and clinical features of the subjects were listed in Additional file 1: Table 1. This research included 315 COPD patients (239 males and 76 females) and 314 healthy controls (177 males and 137 females). The mean age of the case and control group was 71.23 ± 6.83 and 71.93 ± 10.11 years, respectively. Besides, there was no significant difference in age (*p* = 0.306) and gender (*p* = 0.926) distribution between the two groups.

### Evaluation of COPD risk

The detailed information of SNPs in *FGFR2* and *MGAT5* is summarized in Table 1. HaploReg v4.1 showed that *FGFR2* and *MGAT5* SNPs were associated with the regulation of SiPhy cons, Enhancer histone marks, DNAse, Motifs changed, Selected eQTL hits, GRASP QTL hits, and Promoter histone marks. All SNPs conformed to HWE (*p* > 0.05). Our results showed that the A allele of rs2420915 near *FGFR2* increased the risk of COPD (OR 1.41, 95% CI 1.12–1.77, *p* = 0.004). However, the A allele of rs6430491near *MGAT5* (OR 0.69, 95% CI 0.55–0.87, *p* = 0.002) and the G allele of *MGAT5*-rs2593704 (OR 0.74, 95% CI 0.57–0.95, *p* = 0.020) were correlated to reduced risk of COPD.

**Table 1:** The primary information of SNPs in *FGFR2* and *MGAT5*

SNP	Gene	Chr	Position	Allele A/B	Location	MAF		HWE *p*	OR (95% CI)	*p*	HaploReg
						Case	Control				
rs2420915	*FGFR2*	10	122840277	A/G	Near	0.414	0.334	0.801	1.41 (1.12–1.77)	**0.004**	SiPhy cons, Enhancer histone marks, DNAse
rs1907240	*FGFR2*	10	122897959	G/A	Intron	0.423	0.387	0.905	1.16 (0.93–1.46)	0.190	SiPhy cons, Enhancer histone marks, DNAse, Motifs changed, Selected eQTL hits
rs2257129	*FGFR2*	10	122898697	T/C	Intron	0.414	0.387	0.722	1.12 (0.89–1.40)	0.344	Enhancer histone marks, Motifs changed
rs6430491	*MGAT5*	2	134840967	A/G	Near	0.351	0.438	0.909	0.69 (0.55–0.87)	**0.002**	Enhancer histone marks, Motifs changed, GRASP QTL hits
rs2593704	*MGAT5*	2	135005277	G/C	Intron	0.217	0.274	0.887	0.74 (0.57–0.95)	**0.020**	Promoter histone marks, Enhancer histone marks, DNAse, Proteins bound

*SNP* single nucleotide polymorphism, *MAF* minor allele frequency, *HWE* Hardy–Weinberg equilibrium, *OR* odds ratio, *95% CI* 95% confidence interval

*p* values were calculated from χ^2^ test

Bold values represent statistical significance (*p* < 0.05)

The relationship between SNPs and COPD risk was assessed in four genetic models. As presented in Table 2, rs2420915 was associated with a higher risk of COPD in codominant (AA: OR 1.85, 95% CI 1.12–3.07, *p* = 0.016; AG: OR 1.62, 95% CI 1.15–2.28, *p* = 0.006), dominant (OR 1.67, 95% CI 1.20–2.31, *p* = 0.002), and additive models (OR 1.43, 95% CI 1.13–1.81, *p* = 0.003). Rs6430491 decreased the susceptibility of COPD in codominant (OR 0.41, 95% CI 0.25–0.68, *p* = 0.0005), dominant (OR 0.70, 95% CI 0.50–0.97, *p* = 0.033), recessive (OR 0.47, 95% CI 0.29–0.74, *p* = 0.0012), and additive models (OR 0.68, 95% CI 0.54–0.86, *p* = 0.0012). While *MGAT5*-rs2593704 reduced the risk of COPD only in dominant (OR 0.70, 95% CI 0.51–0.97, *p* = 0.031) and additive models (OR 0.75, 95% CI 0.58–0.97, *p* = 0.029).

**Table 2:** Associations of *FGFR2* and *MGAT5* genetic variants with COPD risk

Gene	SNP	Model	Genotype	Frequency		Without adjustment		With adjustment	
				Case	Control	OR (95% CI)	*p*^*a*^	OR (95% CI)	*p*^*b*^
*FGFR2*	rs2420915	Codominant	GG	0.326	0.446	1.00		1.00	
			AA	0.153	0.115	1.83 (1.11–3.02)	**0.018**	1.85 (1.12–3.07)	**0.016**
			AG	0.521	0.439	1.62 (1.15–2.28)	0.006	1.62 (1.15–2.28)	**0.006**
		Dominant	GG	0.326	0.446	1.00		1.00	
			AA + AG	0.674	0.554	1.66 (1.20–2.30)	**0.002**	1.67 (1.20–2.31)	**0.002**
		Recessive	AG + GG	0.847	0.885	1.00		1.00	
			AA	0.153	0.115	1.40 (0.88–2.22)	0.156	1.42 (0.89–2.26)	0.139
		Additive	–	–	–	1.42 (1.12–1.80)	**0.003**	1.43 (1.13–1.81)	**0.003**
*FGFR2*	rs1907240	Codominant	AA	0.348	0.373	1.00		1.00	
			GG	0.195	0.146	1.42 (0.90–2.26)	0.135	1.43 (0.90–2.27)	0.133
			GA	0.457	0.481	1.02 (0.72–1.44)	0.926	1.02 (0.72–1.45)	0.901
		Dominant	AA	0.348	0.373	1.00		1.00	
			GG + GA	0.652	0.627	1.11 (0.80–1.54)	0.525	1.12 (0.81–1.55)	0.507
		Recessive	GA + AA	0.805	0.854	1.00		1.00	
			GG	0.195	0.146	1.41 (0.93–2.15)	0.108	1.41 (0.93–2.15)	0.109
		Additive	–	–	–	1.16 (0.93–1.45)	0.196	1.16 (0.93–1.45)	0.190
*FGFR2*	rs2257129	Codominant	CC	0.342	0.370	1.00		1.00	
			TT	0.169	0.145	1.27 (0.79–2.04)	0.332	1.27 (0.79–2.05)	0.329
			TC	0.489	0.486	1.09 (0.77–1.54)	0.629	1.10 (0.77–1.55)	0.607
		Dominant	CC	0.342	0.370	1.00		1.00	
			TT + TC	0.658	0.630	1.13 (0.81–1.57)	0.466	1.14 (0.82–1.58)	0.450
		Recessive	TC + CC	0.831	0.855	1.00		1.00	
			TT	0.169	0.145	1.21 (0.78–1.86)	0.398	1.20 (0.78–1.86)	0.402
		Additive	–			1.12 (0.89–1.40)	0.340	1.12 (0.89–1.41)	0.333
*MGAT5*	rs6430491	Codominant	GG	0.400	0.318	1.00		1.00	
			AA	0.102	0.194	0.42 (0.25–0.69)	**0.0006**	0.41 (0.25–0.68)	**0.0005**
			AG	0.498	0.487	0.81 (0.58–1.15)	0.242	0.81 (0.58–1.15)	0.240
		Dominant	GG	0.400	0.318	1.00		1.00	
			AA + AG	0.600	0.682	0.70 (0.51–0.97)	**0.033**	0.70 (0.50–0.97)	**0.033**
		Recessive	AG + GG	0.898	0.806	1.00		1.00	
			AA	0.102	0.194	0.47 (0.30–0.74)	**0.0012**	0.47 (0.29–0.74)	**0.0012**
		Additive	–	–	–	0.68 (0.54–0.86)	**0.0014**	0.68 (0.54–0.86)	**0.0013**
*MGAT5*	rs2593704	Codominant	CC	0.619	0.529	1.00		1.00	
			GG	0.054	0.077	0.60 (0.31–1.15)	0.121	0.61 (0.31–1.17)	0.136
			GC	0.327	0.394	0.71 (0.51–0.99)	**0.045**	0.72 (0.51–1.01)	0.057
		Dominant	CC	0.619	0.529	1.00		1.00	
			GG + GC	0.381	0.471	0.69 (0.50–0.95)	**0.023**	0.70 (0.51–0.97)	**0.031**
		Recessive	GC + CC	0.946	0.923	1.00		1.00	
			GG	0.054	0.077	0.68 (0.36–1.29)	0.239	0.69 (0.36–1.31)	0.258
		Additive	–	–	–	0.74 (0.57–0.96)	**0.022**	0.75 (0.58–0.97)	**0.029**

*SNP* single nucleotide polymorphism, *OR* odds ratio, *95% CI* 95% confidence interval

*p*^a^ values were calculated by logistic regression analysis with the comparison between COPD patients and healthy controls

*p*^b^ values were calculated by logistic regression analysis with adjustment for age and gender

Bold values indicate statistical significance (*p* < 0.05)

Next, we evaluated the association of *FGFR2* and *MGAT5* variants with COPD susceptibility in different subgroups (Tables 3, 4, 5). Rs2420915 promoted the development of COPD in men, women, non-smokers, and individuals older than 70 years (*p* < 0.05). *FGFR2-*rs1907240, and -rs2257129 augmented the likelihood of COPD in non-smokers (*p* < 0.05). Rs6430491 in non-smokers and subjects in BMI ≤ 24 kg/m^2^ and rs2593704 in males, smokers, and individuals aged < 70 years and BMI ≤ 24 kg/m^2^ decreased the occurrence of COPD (*p* < 0.05).

**Table 3:** The relationship of *FGFR2* and *MGAT5* SNPs with COPD risk stratified by age and gender

Age										
Gene SNP	Model	Genotype	> 70 years				≤ 70 years			
			Frequency in case	Frequency in control	OR (95% CI)	*p*	Frequency in case	Frequency in control	OR(95% CI)	*p*
*FGFR* rs2420915	Allele	G	0.581	0.658	1.00		0.594	0.675	1.00	
		A	0.419	0.342	1.39 (1.03–1.88)	**0.032**	0.406	0.325	1.42 (0.99–2.02)	0.054
	Codominant	GG	0.301	0.429	1.00		0.362	0.467	1.00	
		AA	0.140	0.113	2.09 (1.03–4.25)	**0.043**	0.173	0.117	1.79 (0.83–3.86)	0.137
		AG	0.559	0.458	1.87 (1.17–3.01)	**0.010**	0.465	0.416	1.41 (0.83–2.42)	0.207
	Dominant	GG	0.301	0.429	1.00		0.362	0.467	1.00	
		AA + AG	0.699	0.571	1.91 (1.21–3.02)	**0.005**	0.638	0.533	1.50 (0.90–2.48)	0.119
	Recessive	AG + GG	0.860	0.887	1.00		0.827	0.883	1.00	
		AA	0.140	0.113	1.43 (0.75–2.74)	0.278	0.173	0.117	1.50 (0.73–3.06)	0.269
	Additive	–	–	–	1.55 (1.11–2.17)	0.010	–	–	1.36 (0.95–1.95)	0.097
*MGAT5* rs2593704	Allele	C	0.803	0.766	1.00		0.752	0.675	1.00	
		G	0.197	0.234	0.80 (0.56–1.14)	0.223	0.248	0.325	0.69 (0.47–1.00)	0.052
	Codominant	CC	0.654	0.601	1.00		0.567	0.438	1.00	
		GG	0.048	0.069	0.72 (0.28–1.83)	0.490	0.063	0.088	0.53 (0.20–1.43)	0.212
		GC	0.298	0.330	0.88 (0.55–1.41)	0.599	0.370	0.474	0.60 (0.36–1.01)	0.054
	Dominant	CC	0.654	0.601	1.00		0.567	0.438	1.00	
		GG + GC	0.346	0.399	0.85 (0.55–1.33)	0.487	0.433	0.562	0.59 (0.36–0.97)	**0.038**
	Recessive	GC + CC	0.952	0.931	1.00		0.937	0.912	1.00	
		GG	0.048	0.069	0.75 (0.30–1.89)	0.541	0.063	0.088	0.68 (0.26–1.76)	0.423
	Additive	–	–	–	0.86 (0.60–1.24)	0.425	–	–	0.67 (0.44–0.99)	**0.047**

Gender										
Gene SNP	Model	Genotype	Male				Female			
			Frequency in case	Frequency in control	OR (95% CI)	*p*	Frequency in case	Frequency in control	OR (95% CI)	*p*
FGFR	Allele	G	0.601	0.667	1.00		0.540	0.662	1.00	
rs2420915		A	0.399	0.333	1.33 (1.02–1.73)	**0.035**	0.460	0.338	1.67 (1.05–2.66)	**0.029**
	Codominant	GG	0.344	0.451	1.00		0.267	0.429	1.00	
		AA	0.143	0.119	1.63 (0.91–2.90)	0.101	0.187	0.104	2.91 (1.04–8.17)	**0.043**
		AG	0.513	0.430	1.55 (1.05–2.30)	**0.027**	0.546	0.467	1.90 (0.93–3.88)	**0.080**
	Dominant	GG	0.344	0.451	1.00		0.267	0.429	1.00	
		AA + AG	0.656	0.549	1.57 (1.08–2.27)	**0.017**	0.733	0.571	2.08 (1.05–4.13)	**0.036**
	Recessive	AG + GG	0.857	0.881	1.00		0.813	0.896	1.00	
		AA	0.143	0.119	1.28 (0.75–2.19)	0.370	0.187	0.104	1.98 (0.78–5.04)	0.152
	Additive	–	–	–	1.35 (1.03–1.77)	**0.030**	–	–	1.75 (1.07–2.86)	**0.026**
*MGAT5*	Allele	C	0.795	0.714	0.64 (0.48–0.87)	**0.004**	0.743	0.764	1.11 (0.66–1.89)	0.687
rs2593704		G	0.205	0.286	1.00		0.257	0.236	1.00	
	Codominant	CC	0.645	0.508	0.54 (0.26–1.15)	0.110	0.539	0.595	0.86 (0.22–3.44)	0.832
		GG	0.054	0.081	0.59 (0.40–0.87)	**0.008**	0.053	0.068	1.33 (0.68–2.62)	0.407
		GC	0.301	0.411	1.00		0.408	0.337	1.00	
	Dominant	CC	0.645	0.508	0.58 (0.40–0.85)	**0.004**	0.539	0.595	1.25 (0.66–2.40)	0.494
		GG + GC	0.355	0.492	1.00		0.461	0.405	1.00	
	Recessive	GC + CC	0.946	0.919	0.67 (0.32–1.39)	0.279	0.947	0.932	0.77 (0.20–2.99)	0.703
		GG	0.054	0.081	0.66 (0.49–0.89)	**0.007**	0.053	0.068	1.12 (0.66–1.89)	0.683
	Additive	–	–	–	0.64 (0.48–0.87)	**0.004**	–	–	1.11 (0.66–1.89)	0.687

*SNP* single nucleotide polymorphism, *OR* odds ratio, *95% CI* 95% confidence interval

*p* values were calculated by logistic regression analysis with adjustment for age and gender

Bold values indicate statistical significance (*p* < 0.05)

**Table 4:** The relationship of *FGFR2* and *MGAT5* SNPs with COPD risk stratified by smoking

Gene SNP	Model	Genotype	Smoking				No smoking			
			Frequency in case	Frequency in control	OR (95% CI)	*p*	Frequency in case	Frequency in control	OR(95% CI)	*p*
*FGFR2* rs2420915	Allele	G	0.613	0.615	1.00		0.567	0.674	1.00	
		A	0.387	0.385	1.01 (0.64–1.60)	0.966	0.433	0.326	1.58 (1.11–2.24)	**0.010**
	Codominant	GG	0.370	0.385	1.00		0.291	0.458	1.00	
		AA	0.144	0.154	1.07 (0.40–2.87)	0.894	0.158	0.110	2.16 (0.99–4.68)	0.052
		AG	0.486	0.461	1.11 (0.55–2.23)	0.781	0.551	0.432	1.96 (1.16–3.29)	**0.012**
	Dominant	GG	0.370	0.385	1.00		0.291	0.458	1.00	
		AA + AG	0.630	0.615	1.10 (0.57–2.12)	0.785	0.709	0.542	2.00 (1.21–3.28)	**0.006**
	Recessive	AG + GG	0.856	0.846	1.00		0.842	0.890	1.00	
		AA	0.144	0.154	1.01 (0.41–2.53)	0.977	0.158	0.110	1.46 (0.72–3.00)	0.297
	Additive	–	–	–	1.05 (0.66–1.68)	0.836	–	–	1.60 (1.10–2.31)	**0.013**
*FGFR2* rs1907240	Allele	A	0.617	0.577	1.00		0.542	0.640	1.00	
		G	0.383	0.423	0.85 (0.54–1.33)	0.470	0.458	0.360	1.50 (1.07–2.11)	**0.020**
	Codominant	AA	0.407	0.327	1.00		0.301	0.381	1.00	
		GG	0.172	0.173	0.92 (0.35–2.40)	0.859	0.217	0.102	2.63 (1.22–5.68)	**0.014**
		GA	0.421	0.500	0.68 (0.33–1.38)	0.282	0.482	0.517	1.19 (0.70–2.01)	0.525
	Dominant	AA	0.407	0.327	1.00		0.301	0.381	1.00	
		GG + GA	0.593	0.673	0.73 (0.37–1.44)	0.366	0.699	0.619	1.43 (0.86–2.35)	0.165
	Recessive	GA + AA	0.828	0.827	1.00		0.783	0.898	1.00	
		GG	0.172	0.173	1.14 (0.48–2.72)	0.769	0.217	0.102	2.38 (1.17–4.81)	**0.016**
	Additive	–	–	–	0.90 (0.58–1.41)	0.645	–	–	1.51 (1.06–2.14)	**0.023**
*FGFR2* rs2257129	Allele	C	0.627	0.577	1.00		0.552	0.640	1.00	
		T	0.373	0.423	0.81 (0.52–1.28)	0.371	0.448	0.360	1.45 (1.03–2.04)	**0.035**
	Codominant	CC	0.390	0.327	1.00		0.303	0.381	1.00	
		TT	0.137	0.173	0.75 (0.28–2.02)	0.572	0.200	0.102	2.40 (1.11–5.23)	**0.027**
		TC	0.473	0.500	0.79 (0.39–1.61)	0.517	0.497	0.517	1.21 (0.72–2.05)	0.468
	Dominant	CC	0.390	0.327	1.00		0.303	0.381	1.00	
		TT + TC	0.610	0.673	0.78 (0.40–1.54)	0.474	0.697	0.619	1.41 (0.86–2.33)	0.177
	Recessive	TC + CC	0.863	0.827	1.00		0.800	0.898	1.00	
		TT	0.137	0.173	0.86 (0.35–2.11)	0.742	0.200	0.102	2.14 (1.05–4.36)	**0.036**
	Additive	–	–	–	0.85 (0.53–1.36)	0.499	–	–	1.46 (1.02–2.09)	**0.039**
*MGAT5* rs6430491	Allele	G	0.588	0.606	1.00		0.699	0.576	1.00	
		A	0.412	0.394	1.08 (0.68–1.70)	0.757	0.301	0.424	0.59 (0.41–0.83)	**0.003**
	Codominant	GG	0.333	0.365	1.00		0.452	0.364	1.00	
		AA	0.156	0.154	1.11 (0.42–2.91)	0.839	0.054	0.212	0.21 (0.09–0.49)	**0.0003**
		AG	0.511	0.481	1.28 (0.63–2.62)	0.495	0.494	0.424	0.92 (0.55–1.55)	0.758
	Dominant	GG	0.333	0.365	1.00		0.452	0.364	1.00	
		AA + AG	0.667	0.635	1.24 (0.63–2.42)	0.537	0.548	0.636	0.69 (0.42–1.12)	0.136
	Recessive	AG + GG	0.844	0.846	1.00		0.946	0.788	1.00	
		AA	0.156	0.154	0.96 (0.40–2.32)	0.927	0.054	0.212	0.22 (0.10–0.49)	**0.0002**
	Additive	–	–	–	1.10 (0.68–1.76)	0.704	–	–	0.58 (0.40–0.83)	**0.003**
*MGAT5* rs2593704	Allele	C	0.789	0.635	1.00		0.777	0.770	1.00	
		G	0.211	0.365	0.46 (0.29–0.76)	**0.002**	0.223	0.230	0.96 (0.64–1.43)	0.834
	Codominant	CC	0.639	0.404	1.00		0.602	0.609	1.00	
		GG	0.061	0.135	0.37 (0.12–1.16)	0.088	0.048	0.070	0.73 (0.26–2.04)	0.544
		GC	0.300	0.461	0.42 (0.21–0.84)	**0.014**	0.350	0.321	1.09 (0.65–1.82)	0.756
	Dominant	CC	0.639	0.404	1.00		0.602	0.609	1.00	
		GG + GC	0.361	0.596	0.41 (0.21–0.79)	**0.008**	0.398	0.391	1.02 (0.63–1.67)	0.930
	Recessive	GC + CC	0.939	0.865	1.00		0.952	0.930	1.00	
		GG	0.061	0.135	0.54 (0.18–1.62)	0.268	0.048	0.070	0.71 (0.26–1.95)	0.501
	Additive	–	–	–	0.53 (0.32–0.87)	**0.012**	–	–	0.96 (0.65–1.43)	0.850

*SNP* single nucleotide polymorphism, *OR* odds ratio, *95% CI* 95% confidence interval

*p* values were calculated by logistic regression analysis with adjustment for age and gender

Bold values indicate statistical significance (*p* < 0.05)

**Table 5:** The relationship of *FGFR2* and *MGAT5* SNPs with COPD risk stratified by BMI

Gene	SNP	Model	Genotype	BMI ≤ 24			
				Frequency in case	Frequency in control	OR (95% CI)	*p*
*FGFR2*	rs2420915	Allele	G	0.586	0.627	1.00	
			A	0.414	0.373	1.19 (0.80–1.76)	0.392
		Codominant	GG	0.320	0.373	1.00	
			AA	0.148	0.120	1.58 (0.64–3.92)	0.324
			AG	0.532	0.507	1.19 (0.65–2.17)	0.574
		Dominant	GG	0.320	0.373	1.00	
			AA + AG	0.680	0.627	1.26 (0.71–2.24)	0.429
		Recessive	AG + GG	0.852	0.880	1.00	
			AA	0.148	0.120	1.43 (0.62–3.31)	0.406
		Additive	–	–	–	1.24 (0.81–1.88)	0.318
*FGFR2*	rs1907240	Allele	A	0.574	0.597	1.00	
			G	0.426	0.403	1.10 (0.75–1.62)	0.632
		Codominant	AA	0.348	0.328	1.00	
			GG	0.200	0.135	1.43 (0.60–3.41)	0.419
			GA	0.452	0.537	0.79 (0.43–1.46)	0.452
		Dominant	AA	0.348	0.328	1.00	
			GG + GA	0.652	0.672	0.92 (0.51–1.64)	0.771
		Recessive	GA + AA	0.800	0.865	1.00	
			GG	0.200	0.135	1.64 (0.75–3.60)	0.215
		Additive	–	–	–	1.10 (0.75–1.63)	0.626
*FGFR2*	rs2257129	Allele	C	0.586	0.597	1.00	
			T	0.414	0.403	1.05 (0.71–1.54)	0.824
		Codominant	CC	0.341	0.328	1.00	
			TT	0.169	0.135	1.23 (0.51–2.97)	0.641
			TC	0.490	0.537	0.86 (0.47–1.59)	0.641
		Dominant	CC	0.341	0.328	1.00	
			TT + TC	0.659	0.672	0.94 (0.52–1.68)	0.827
		Recessive	TC + CC	0.831	0.865	1.00	
			TT	0.169	0.135	1.35 (0.61–2.99)	0.466
		Additive	–	–	–	1.05 (0.70–1.57)	0.811
*MGAT5*	rs6430491	Allele	G	0.639	0.604	1.00	
			A	0.361	0.396	0.86 (0.58–1.27)	0.456
		Codominant	GG	0.386	0.403	1.00	
			AA	0.108	0.194	0.48 (0.21–1.08)	0.075
			AG	0.506	0.403	1.19 (0.65–2.20)	0.572
		Dominant	GG	0.386	0.403	1.00	
			AA + AG	0.614	0.597	0.96 (0.54–1.69)	0.888
		Recessive	AG + GG	0.892	0.806	1.00	
			AA	0.108	0.194	0.43 (0.20–0.920	0.029
		Additive	–	–	–	0.78 (0.51–1.18)	0.231
*MGAT5*	rs2593704	Allele	C	0.791	0.657	1.00	
			G	0.209	0.343	0.51 (0.33–0.77)	**0.001**
		Codominant	CC	0.633	0.433	1.00	
			GG	0.052	0.119	0.31 (0.12–0.83)	**0.020**
			GC	0.315	0.448	0.52 (0.29–0.94)	**0.031**
		Dominant	CC	0.633	0.433	1.00	
			GG + GC	0.367	0.567	0.48 (0.27–0.83)	**0.009**
		Recessive	GC + CC	0.948	0.881	1.00	
			GG	0.052	0.119	0.41 (0.16–1.05)	0.064
		Additive	–	–	–	0.54 (0.36–0.83)	**0.005**

*SNP* single nucleotide polymorphism, *OR* odds ratio, *95% CI* 95% confidence interval

*p* values were calculated by logistic regression analysis with adjustment for age and gender

Bold values indicate statistical significance (*p* < 0.05)

### Haplotype analysis

**Fig. 1:**
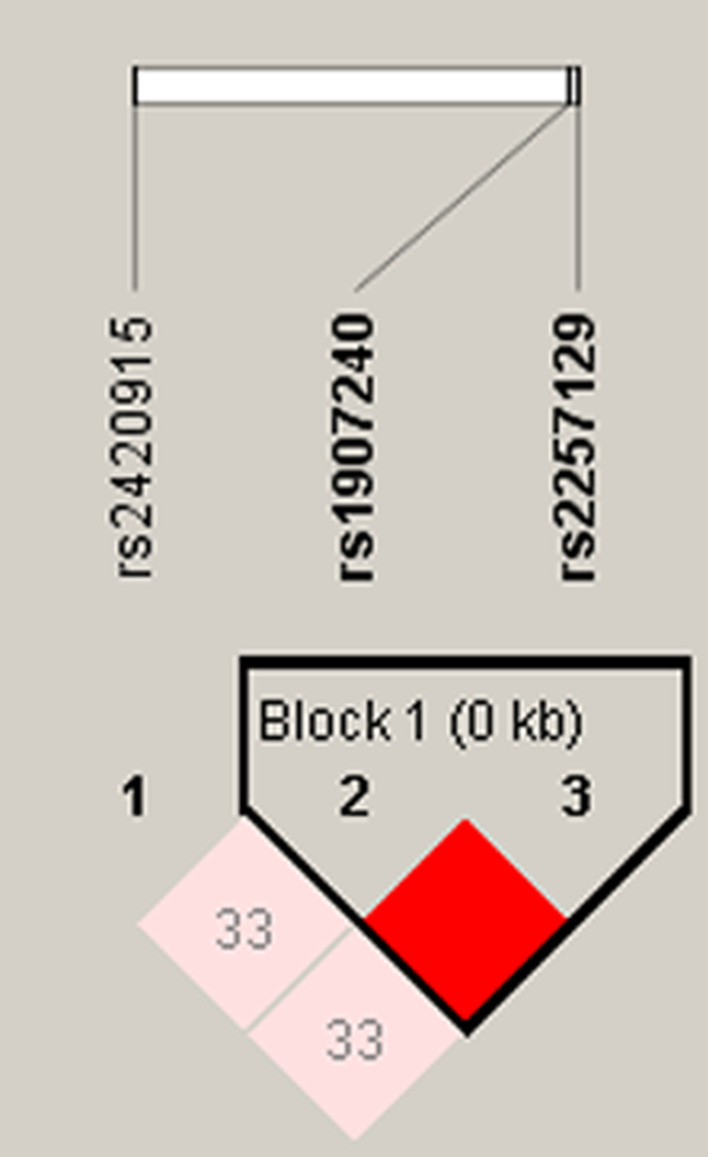
Haplotype block map for SNPs of *FGFR2.* Block 1 includes rs1907240, and rs2257129. The numbers inside the diamonds indicate the D’ for pairwise analyses

We further analyzed the haplotype and linkage disequilibrium of *FGFR2* and *MGAT5* variants in cases and control group. The results in Fig. 1 showed that an LD plot consisted of two SNPs (rs1907240 and rs2257129). And there was no correlation of haplotypes with COPD risk (*p* > 0.05, Table 6).

**Table 6:** Haplotype association of *FGFR2* polymorphisms with COPD risk

SNP	Haplotype	Frequency in cases	Frequency in controls	Without adjustment		With adjustment	
				OR (95% CI)	*p*	OR (95% CI)	*p*
rs1907240|rs2257129	GT	0.410	0.388	1.10 (0.88–1.38)	0.416	1.10 (0.88–1.38)	0.409
rs1907240|rs2257129	AC	0.423	0.388	1.16 (0.92–1.45)	0.207	1.16 (0.92–1.45)	0.203

*SNP* single nucleotide polymorphism, *OR* odds ratio, *CI* confidence interval

## Discussion

We assessed the correlation between *FGFR2* and *MGAT5* mutants and COPD susceptibility in the Chinese population. The results revealed that rs2420915 increased the incidence of COPD, while rs6430491, rs2593704 reduced the risk of COPD. In addition, rs2420915, rs1907240, rs2257129, rs6430491, and rs2593704 were associated with COPD susceptibility in different subgroups. These data emphasized the crucial role of *FGFR2* and *MGAT5* in the pathogenesis of COPD, and provide new biomarkers for the treatment and diagnosis of COPD.

The *FGFR2* gene belongs to the fibroblast growth factor receptor family and is located on chromosome 10q26.13 in humans. *FGFR2* gene has been reported to encode *FGFR2b* in epithelial cells and *FGFR2c* in mesenchymal cells [23]. Yu et al. [24] have found that *FGFR2* mutant attenuated lung fibrosis by inhibiting α-smooth muscle actin and collagen deposit. Furthermore, Masunaga et al. [25] indicated that the expression of *FGFR2* was highly expressed in pulmonary papillary adenoma cells compared with nontumorous lung. Besides, *FGFR2b* signaling facilitated alveolar epithelial regeneration through bronchial epithelial stem cells after lung injury [26]. These findings demonstrated that *FGFR2* gene played a crucial role in lung disease. However, there are no reports on rs2420915, rs1907240, and rs2257129 in lung disease and COPD. In our study, we first investigated the impact of rs2420915, rs1907240, rs2257129 on the occurrence of COPD. The results indicated that rs2420915*, FGFR2-*rs1907240, and -rs2257129 were risk factors for COPD development. These data suggested that *FGFR2* variants may be involved in COPD development, and it provided new clues for individualized treatment of COPD patients.

*MGAT5*, a typical cancer-associated glycosyltransferase, is located in 2q21.2-q21.3. It is closely associated with the growth, migration, and invasion of tumor cells [27, 28]. Dosaka-Akita et al. [29] found that *MGAT5* is associated with histology and prognosis in non-small cell lung cancers. Similarly, Zhou et al. [18] reported that *MGAT5* was overexpressed in pulmonary adenocarcinoma cells, and knockdown of M*GAT5* could suppress cell growth both in vitro and in vivo. Moreover, Elek et al. [20] demonstrated that the allele frequencies of rs34944508 in the 3′-UTR of *MGAT5* gene were significantly different among control, COPD, lung cancer, and comorbid COPD and lung cancer, and indicated that rs34944508 might influence lung cancer risk in Caucasian. Nevertheless, no studies focused on the role of rs6430491, and rs2593704 in COPD development. We, for the first time, found rs6430491, and *MGAT5*-rs2593704 are correlated with a decreased risk of COPD, and illustrated that *MGAT5* gene has a potential role in the pathogenesis of COPD.

Some research has shown that the intronic SNPs can modify gene function by altering the expression of gene [30, 31]. In our research, rs1907240, rs2257129, rs2593704 are located in the intron region of *FGFR2* and *MGAT5* gene*.* Combining previous studies and database predictions, we hypothesize that *FGFR2* and *MGAT5* intron SNPs cause changes in *FGFR2* and *MGAT5* expression and activity via influencing mRNA splicing and ultimately affect disease susceptibility. In subsequent experiments, we will examine the functional consequences of the intronic polymorphisms to support our hypothesis in vitro and ex vivo, focusing on the regulation of gene expression and splicing. In addition, rs2420915 and rs6430491 were associated with the regulation of SiPhy cons, enhancer histone marks, DNAse, motifs changed, GRASP QTL hits. These functions could affect the expression of gene, and ultimately alter the susceptibility of COPD.

Although the interesting results on the relationship of *FGFR2* and *MGAT5* polymorphisms with COPD susceptibility, several limitations of this study need to be stated. Firstly, we only genotyped three SNPs in *FGFR2* and two SNPs in *MGAT5*, more SNPs of these two genes are needed to investigate. Secondly, the selection bias is inevitable when all the study individuals are enrolled from the same hospital. Thirdly, the molecular mechanism of *FGFR2* and *MGAT5* to COPD susceptibility remains unknown and should be studied in further study.

## Conclusions

Our results suggested that *FGFR2* and *MGAT5* genetic polymorphisms are correlated with the risk of COPD in the Chinese Han people. These data underscored the important role of *FGFR2* and *MGAT5* gene in the occurrence of COPD and provided new biomarkers for COPD treatment.

## Supplementary Information

**Additional file 1: Supplemental table 1.** Demographic and clinical characteristics of study populations.

## Data Availability

All data generated or analyzed during this study are included in this published article.

## Abbreviations

COPD: Chronic obstructive pulmonary disease
FEV_1_: Forced expiratory volume in one second
FVC: Forced vital capacity
BMI: Body mass index
SNP: Single nucleotide polymorphism
HWE: Hardy–Weinberg equilibrium
OR: Odd ratio
CI: 95% Confidence intervals
FGFR2: Fibroblast growth factor receptor 2
MGAT5: N-acetylglucosaminyltransferaseV
