# Supplementary material for: The polymorphisms of FGFR2 and MGAT5 affect the susceptibility to COPD in the Chinese people
Source: BMC Pulm Med. 2021 Apr 20;21:129. doi: 10.1186/s12890-021-01498-3 (PMC8058990; doi:10.1186/s12890-021-01498-3)
Supplement: Supplementary file 1 — Additional file 1: Supplemental table 1. Demographic and clinical characteristics of study populations. [file 12890_2021_1498_MOESM1_ESM.docx]

**The polymorphisms of** ***FGFR2* and *MGAT5* affect the susceptibility to COPD in the Chinese people**

Running Title: ***FGFR2* and *MGAT5* influence COPD risk**

Xiaobo Li^1^**^#^**, Guangyu Zhou^2^**^#^**, Fei Chen^3,5^**^#^**, Xiaobo Tian^4^, Guoyao Li^1^, Yipeng Ding^6*^

1. Department of General Practice, People’s Hospital of Wanning, Wanning 571500, Hainan, China

2. Department of Nursing, People’s Hospital of Wanning, Wanning 571500, Hainan, China

3. Nanyang branch of Wencheng health center of Wenchang City, Wenchang 571399, Hainan, China

1. Department of Medical, People’s Hospital of Wanning, Wanning 571500, Hainan, China
2. Department of Science and Education Department, Hainan General Hospital, Hainan affiliated Hospital of Hainan Medical University, Haikou 570311, Hainan, China
3. Department of General Practice, Hainan General Hospital, Hainan affiliated Hospital of Hainan Medical University, Haikou 570311, Hainan, China

^#^ Xiaobo Li, Guangyu Zhou and Fei Chen contributed equally to this work.

***Corresponding author**

**Yipeng Ding**

**E-mail:** ypding@yeah.net

**Tel:** +86-18976335858

**Address:** No. 19, Xinhua Road, Xiuying District, Haikou570311, Hainan, China

Supplemental table 1 Demographic and clinical characteristics of study populations

| Variable | COPD patients  (N=315) | Controls  (N=314) | *p* |
| --- | --- | --- | --- |
| Age (years) | 71.23 ± 6.83 | 71.93 ± 10.11 | 0.306^a^ |
| > 70 | 188(60%) | 177(56%) |  |
| ≤ 70 | 127(40%) | 137(44%) |  |
| Gender |  |  | 0.926^b^ |
| Men | 239(76%) | 237(75%) |  |
| Women | 76(24%) | 77(25%) |  |
| Smoking |  |  |  |
| Yes | 147(47%) | 52(17%) |  |
| No | 166(53%) | 118(38%) |  |
| Missing | 2 | 144(45%) |  |
| BMI |  |  |  |
| ≤ 24 | 251(80%) | 67(21%) |  |
| > 24 | 29(9%) | 78(25%) |  |
| Missing | 35(11%) | 170(54%) |  |
| Complications |  |  |  |
| Yes | 93(30%) |  |  |
| No | 174(55%) |  |  |
| Missing | 48(15%) |  |  |
| Wheeze |  |  |  |
| Yes | 153(49%) |  |  |
| No | 123(39%) |  |  |
| Missing | 39(12%) |  |  |
| Gasp |  |  |  |
| Yes | 115(37%) |  |  |
| No | 166(53%) |  |  |
| Missing | 34(10%) |  |  |
| Chest distress |  |  |  |
| Yes | 102(32%) |  |  |
| No | 179(57%) |  |  |
| Missing | 34(10%) |  |  |
| Respiratory infection |  |  |  |
| Yes | 164(52%) |  |  |
| No | 117(37%) |  |  |
| Missing | 34(10%) |  |  |

*p*^a^ values were calculated from student’s t test.

*p*^b^ values were calculated from χ^2^ test.

COPD: Chronic obstructive pulmonary disease; BMI, Body mass index.
